# The risk of hospitalization associated with hot nights and excess nighttime heat in a subtropical metropolis: a time-series study in Hong Kong, 2000–2019

**DOI:** 10.1016/j.lanwpc.2024.101168

**Published:** 2024-08-12

**Authors:** Yi Tong Guo, Ka Hung Chan, Hong Qiu, Eliza Lai-yi Wong, Kin Fai Ho

**Affiliations:** a JC School of Public Health and Primary Care, The Chinese University of Hong Kong, Hong Kong SAR, China; b Clinical Trial Service Unit and Epidemiological Studies Unit, Nuffield Department of Population Health, University of Oxford, Oxford, UK; c Oxford British Heart Foundation Centre of Research Excellence, University of Oxford, Oxford, UK; d Institute of Environment, Energy and Sustainability, The Chinese University of Hong Kong, Hong Kong SAR, China

**Keywords:** Climate change, Temperature, Nighttime, Environment, Socioeconomic, Urban

## Abstract

**Background:**

Recent studies showed increased mortality risks after hot nights, but their effect on hospitalizations, especially in vulnerable populations, remains under-studied.

**Methods:**

Daily hospitalization, meteorological (including hourly), and air pollution data were collected for the hot seasons (May–October) of 2000–19 in Hong Kong. We derived three hot-night metrics: HNday_28 °C_, daily minimum temperature ≥28 °C, the governmental definition of hot nights; HNe, hot night excess calculated by summing heat excess of hourly temperatures above 28 °C at night; and HNday_90th_, hot nights classified using the 90th percentile HNe (17.7 °C⋅h) as a cutoff. We fitted time-series regression with distributed lag nonlinear models to examine the associations of hot-night metrics with various hospitalizations.

**Findings:**

During the 3680 study days, 5,002,114 non-cancer non-external (NCNE) hospitalizations were recorded. Half (1874) of the days experienced excess nighttime heat (HNe>0) with a mean (SD) of 8.0 (6.8) °C⋅h; 499 and 187 hot nights were identified by HNday_28 °C_ and HNday_90th_, respectively. Extreme HNe (99th percentile vs 0 °C⋅h) was significantly associated with increased NCNE hospitalizations over lag 0–4 days by 3.1% [95% confidence interval: 1.5%, 4.8%] overall, with enhanced effects in elderly (5.3% [3.2%, 7.4%]), low-SES individuals (5.3% [2.8%, 8.0%]), and circulatory admissions (3.4% [0.2%, 6.8%]). HNday_90th_, reflecting extreme HNe, better identified hazardous hot nights than the official HNday_28 °C_.

**Interpretation:**

Excessive nighttime heat is significantly associated with increased hospitalizations, particularly affecting the elderly and socioeconomically disadvantaged individuals. Nighttime heat intensity should be incorporated in defining hot nights with public health relevance.

**Funding:**

10.13039/501100000274British Heart Foundation.

**Research in context**

**Evidence before this study**

Emerging evidence suggests excess nighttime heat to be associated with risk of mortality independent of daily mean temperature, but little is known about its association with morbidity outcomes. We searched PubMed and Web of Science without language restrictions for studies published from database inception to April 2024, using search terms (“hot night” OR (“night” AND (“high temperature” OR “heat” OR “warming”))) AND (“morbidity” OR “hospitalization” OR “hospital admission” OR “inpatient” OR “outpatient”). Two relevant studies reported somewhat inconsistent findings of nighttime heat association with outpatient visits in China; another study in the USA found weak evidence of association with schizophrenia hospitalization; a few other studies mentioned nighttime heat but provided no direct evidence.

**Added value of this study**

To the best of our knowledge, this is the first study to examine the association of nighttime heat with overall, respiratory, and cardiovascular hospitalization, using 20-year long data from a highly densely populated subtropical metropolis with hot and humid summer (Hong Kong). Nighttime excess heat, measured by hot night excess, was associated with increased risk of hospitalization independent of daily mean temperature, with particularly pronounced associations in elderly and individuals of low socioeconomic status (SES), and on cardiovascular hospitalization. However, local government-defined “hot nights” without concerning nighttime heat intensity was not associated with excess risk.

**Implications of all the available evidence**

Our study shows that nighttime heat could have a significant and independent association with hospitalization risk even in subtropical urban population with supposedly better adaptation to heat. We also highlighted the vulnerability of elderly and people of low SES, informing targeted adaptation empowerment solutions.

## Introduction

Considered as *“the single biggest health threat facing humanity”*, climate change is characterized by increased global average temperature and occurrence and intensity of heatwaves, which are widely known to increase mortality and morbidity.^1^^,^^2^ The rise in average temperature often involves a disproportionate increase in nighttime temperature, especially in high-latitude regions and in urban areas suffering from the urban heat island (UHI) effect.^3^ With the rapid urbanization and industrialization of many low- and middle-income countries, there will be substantially more populations exposed to nighttime heat as global heating worsens.^2^

Nighttime heat could hamper sleep quality and quantity.^4^ Impaired sleep has been linked to a wide range of health issues, such as damaged immunological and metabolic functioning, systemic inflammation, and increased susceptibility to chronic diseases.^5^^,^^6^ However, most previous epidemiological studies focused on average temperature exposure,^7^^,^^8^ whereas studies on the independent health effect of nighttime heat only emerged recently.

Notably, recent studies showed that substantial nighttime heat intensity, typically measured by summing the excess temperature degrees above a threshold during nighttime, was associated with 30–50% and 12–37% higher risks of death in East Asia and Southern Europe, respectively.^9^^,^^10^ However, the association of nighttime heat with morbidity outcomes (e.g., hospitalization) remains understudied.^11^^,^^12^ Mortality studies could only examine the “final” health endpoint, leaving room for reverse causality bias (e.g., incident diseases may induce behavioral changes that alter temperature exposure and thus mortality risks) and unaccounted confounding (e.g., quality of medical treatment, access to healthcare). In contrast, studies of morbidity outcomes could provide clearer insights into the role of nighttime heat on disease incidence or exacerbation and, thus, to inform disease prevention. While heat measured with daily mean temperature has been found to have differing association with mortality and morbidity,^13^ the same is plausible for nighttime heat, and it should be verified. Furthermore, although some studies have investigated the effects of hot nights among different gender, age, and disease groups,^14^, ^15^, ^16^ few have evaluated the vulnerability to nighttime heat in relation to socioeconomic deprivation, which is associated with heightened levels of heat exposure and poor adaptation capacity.^17^

Therefore, using data from Hong Kong, a high-density, subtropical metropolis experiencing a notable rise of hot nights in recent decades, we conducted a time-series study to examine the short–term associations of the intensity of nighttime heat and hot nights with hospitalizations due to different causes, with a special focus on variations in the hospitalization hazards among individuals of different socioeconomic status (SES) and age.

## Methods

### Study population

Hong Kong is a high-density metropolis in southern China, where over seven million residents endure hot and humid subtropical summers along with major UHI effect. During the months of May–October, afternoon temperatures frequently exceed 31 °C, while nighttime temperatures average at ∼26 °C, often accompanied by high humidity.^18^ The city has recorded an average rise of 0.14 °C in annual mean temperature since the late 1900s.^18^ In recent decades, the warming has accelerated, leading to noticeable surges in the occurrence of “very hot days” (i.e., daily maximum temperature ≥33 °C) and hot nights (i.e., daily minimum temperature ≥28 °C) every year.^19^

### Data collection

#### Hospital admission data

Daily counts of hospitalizations via accident and emergency departments of all public hospitals were collected from the Hospital Authority (HA) between 2000 and 2019. Only emergency admissions without a plan were included in this study. As hot nights have only been observed during the hot season, the study period was limited between May and October each year. Due to privacy concerns, a daily count of hospitalization between 1 and 4 was not provided with an accurate number by HA. We hence imputed it as 4 for subsequent analyses.

Three cause/disease groups of interest were extracted given the *International Classification of Diseases, Ninth Revision, Clinical Modification (ICD-9-CM)*, including non-cancer non-external (NCNE; 001–139, 240–799), circulatory (390–459), and respiratory (460–519). NCNE hospitalizations were further classified into three age groups (0–14, 15–64, and 65+), two SES groups (low and high), and six age-SES-specific groups (0–14 low SES, 0–14 high SES, 15–64 low SES, 15–64 high SES, 65+ low SES, and 65+ high SES). A total of fourteen hospitalization series were assessed in this study. Low-SES patients were those receiving a medical fee waiver upon admission, whereas high-SES patients were not receiving the waiver.^20^ Eligible recipients of the waiver scheme were mainly individuals with limited income or assets, including recipients of various social welfare schemes and those belonging to vulnerable groups such as low-income individuals and elderly patients living in poverty.^20^ Their eligibility was reviewed and determined by designated social workers, making it a reliable indicator of individual SES.^20^

#### Meteorological and air pollutant data

Daily data of meteorological factors, including ambient temperatures (in °C; mean, minimum, and maximum), relative humidity (RH, in %), total rainfall (in mm), and wind speed (in km/h), were obtained from the Hong Kong Observatory (HKO) for 2000–2019. Hourly temperature data were also collected for hot-night metrics derivation. Due to its mountainous terrain, the majority of Hong Kong's residents live in high-density areas in relatively close proximity, where the spatial variation of temperature is relatively limited especially in the hot season.^21^ Therefore, the meteorological data were collected from the HKO headquarter station located in the city center, namely the Tsim Sha Tsui area, which have been used to represent the citywide exposure in prior studies.^22^ Furthermore, daily data of four ambient pollutants (in μg/m^3^), including fine particulate matter (PM_2.5_), ozone (O_3_), nitrogen dioxide (NO_2_), and sulphur dioxide (SO_2_), were obtained from the Environmental Protection Department, which administers an air monitoring network comprising fourteen general stations and three roadside stations. Daily mean concentrations of air pollutants were averaged across thirteen general stations, excluding the Tap Mun station which is in a mountain top.

### Statistical analyses

#### Derivation of hot-night metrics

We examined two key metrics that capture different dimensions of hot nights. Officially, the HKO defines a “hot night day” as days with a daily minimum temperature of 28 °C or higher.^19^ Using this governmental definition, we derived a binary indicator HNday_28 °C_, with a value of 1 for days meeting the criteria and 0 for non-HNday_28 °C_. While the study of HNday_28 °C_ allows direct evaluation of the public health relevance of the governmental definition, the binary indicator does not capture the intensity of nighttime heat and the cut-off is mainly based on the distribution of ambient temperatures instead of epidemiological significance. Therefore, as per previous studies,^9^^,^^10^ we computed hot night excess (HNe, in °C⋅h) for each day by summing the extra degrees of hourly temperatures above the 28 °C threshold during nighttime (i.e., 12 h from 20:00 of the previous day [D_t-1_] to 07:59 of the current day [D_t_]). For example, for an individual who experienced 32 °C for 1 h at nighttime, the HNe would be 4 °C⋅h. We chose the above stated timeframe to define nighttime, instead of a meteorological definition (sunset to sunrise), based on a behavior and epidemiology perspective. Specifically, while nighttime heat was hypothesized to be particularly harmful due to its potential to impair rest and sleep, in Hong Kong most individuals would still be engaged in daytime activities well after sunset (generally 17:00–19:00), so inclusion of the early-evening hours could introduce unnecessary noise. Furthermore, the adopted timeframe captured the early-morning, post-sunrise period (05:00–07:00) when daily minimum temperatures typically occur, during which excessive heat may be particularly harmful, as implicated in previous studies.^16^^,^^23^^,^^24^ We also derived an alternative binary definition of hot night days as HNday_90th_, with a value of 1 for days with HNe greater or equal to the 90th percentile of HNe among HNe >0 days (17.7 °C⋅h) and 0 for non-HNday_90th_, which should capture the most hazardous hot nights. Subtypes of HNday_90th_ were also classified given the length of consecutive occurrence (i.e., valued 1 for one day, 2 for two to three days, and 3 for four and more days) and timing of occurrence in the hot season [i.e., valued 1 for early season (May–June), 2 for middle season (July–August), and 3 for late season (September–October)], with non-HNday_90th_ retaining the value of 0.

#### Modeling strategies

We applied quasi-Poisson generalized additive models (GAMs) and distributed lag non-linear models (DLNMs) to examine the short-term risks of hospitalizations with various hot-night metrics.^25^^,^^26^ We first decomposed the hospitalization series to attain stationarity by regressing each outcome on temporal components, i.e., days of the study, days of the week, public holidays, level shifts, and one to two autoregression terms, which were the hospitalization series lagged by 1 or 2 days (Supplementary Method 1: eEquation 1). Smoothing splines were used for days of the study to control trend and seasonality, with numbers of basis per year varying from 1 to 6 depending on outcomes. The optimal choices for decomposition were determined by minimizing quasi Akaike information criterion (AIC) scores and residual partial autocorrelation.^25^ A cross-basis function with a maximum lag of 7 days was created for each hot night metric using DLNMs, which capture the nonlinear and delayed associations of exposure.^26^ For the exposure dimension, linear functions and natural cubic splines with one knot placed at the 50th percentile of the distribution were fit for HNe, while indicator functions were fit for HNday_28 °C_, and HNday_90th_ and its subtypes, respectively; for the lag dimension, quadratic B-splines with two knots equally placed on the log scale of the lag range were applied for all hot-night metrics. We then examined the associations of hospitalizations with the cross-basis terms of hot-night metrics individually, adjusted for temporal components and environmental covariates, including lag 0–3 mean temperatures, RH, total rainfall, wind speed, and PM_2.5_ (see Supplementary Methods 1 for eEquation 2 and justification of adjustment). Lag 0–3 mean temperatures were included to control for the effect of average heat exposure which were found to persist for 3 days in our prior study.^22^ This metric was also less correlated with HNe compared to same-day mean temperatures, thereby reducing the collinearity in the models.

Excess relative risks (ERR) of hospitalizations calculated by (RR-1) × 100%, along with the corresponding 95% confidence intervals (CI), were estimated by comparing (1) HNe at the 99th percentile (28.9 °C⋅h) of the distribution to 0 °C⋅h, (2) HNday_28 °C_ to non-HNday_28 °C_, and (3) HNday_90th_ and its subtypes to non-HNday_90th_, respectively. Of note, we estimated cumulative ERRs over lag 0–4 days because the HNe-hospitalization associations mostly persisted till lag 4 days. Wald tests were performed to examine the difference in ERRs within NCNE age and SES subgroups.^27^

#### Sensitivity analyses

We ran several sensitivity analyses to test the robustness, including estimating cumulative associations over longer lags, changing crossbasis specifications, changing *df* for days of study, and controlling for other air pollutants. We also examined the effect of HNe computed between sunset and sunrise time (obtained from HKO) and compared the results to the main findings.

All statistical analyses were executed in the R platform (version 4.3.0)^28^ using the *mgcv* (version 1.8–42)^25^ and *dlnm* (version 2.4.7)^26^ packages, with a two-tailed significance level of *p* value < 0.05.

#### Role of the funding source

This research did not receive any specific grant.

## Results

### Data summary

**Fig. 1:**
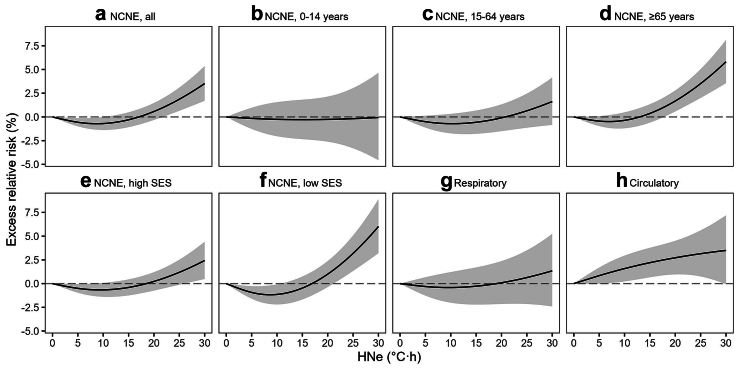
**Cumulative exposure-response associations (lag 0–4 days) of hospitalizations due to a all NCNE, b–d age-specific NCNE, e–f SES-specific NCNE, g respiratory, and h circulatory causes with HNe in Hong Kong, 2000–2019 (May–October)**. HNe was modelled as a nonlinear continuous variable in the crossbasis function. All models were adjusted for trend, seasonality, weekdays, holidays, and daily levels of environmental covariates including mean temperatures averaging over lag 0–3 days, RH, total rainfall, wind speed, and PM_2.5_. Abbreviations: NCNE, non-cancer non-external; RH, relative humidity; PM_2.5_, fine particulate matter; HNe, hot night excess; SES, socioeconomic status.

**Fig. 2:**
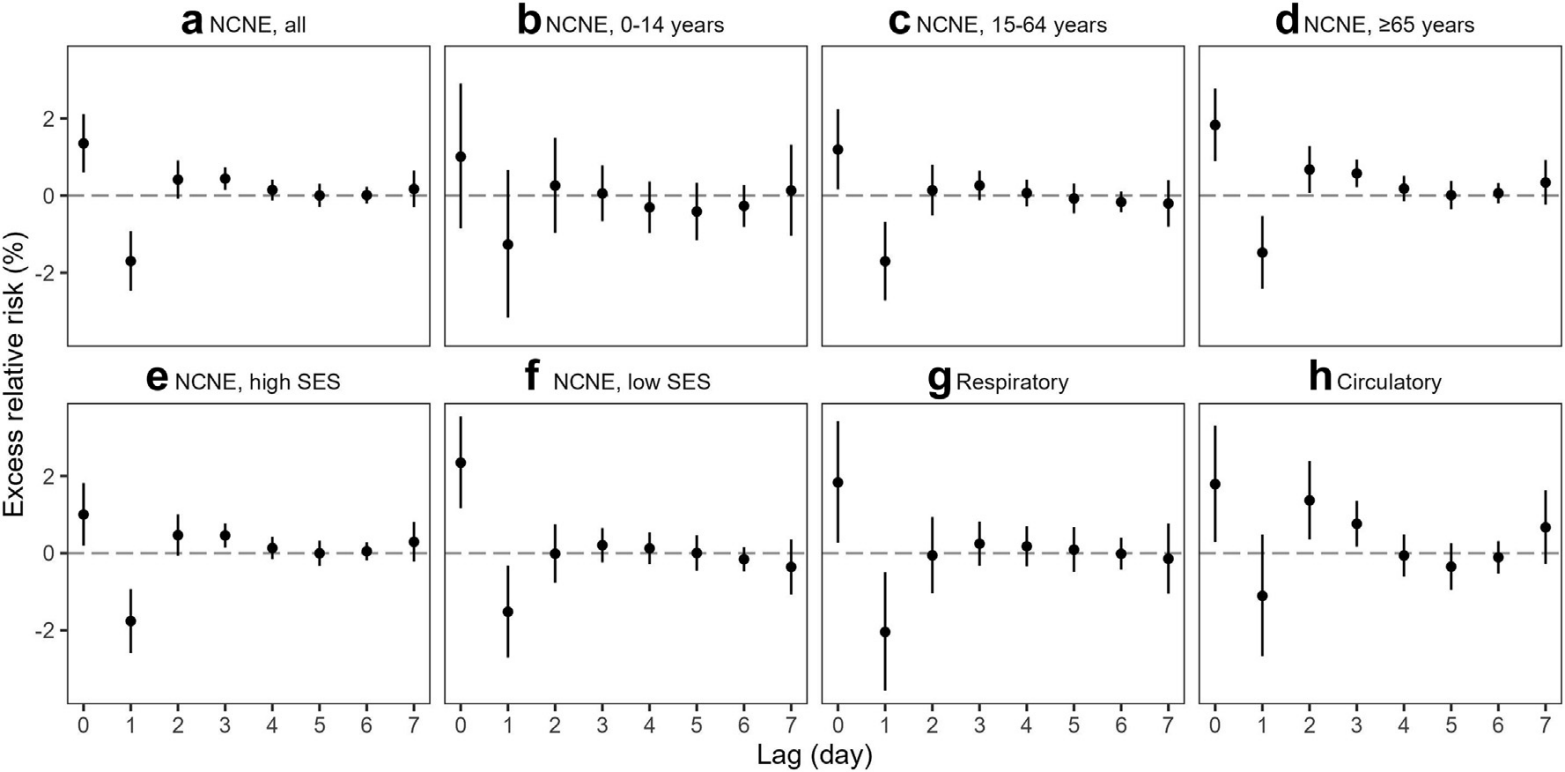
**Lag-response associations of hospitalizations due to a all NCNE, b–d age-specific NCNE, e–f SES-specific NCNE, g respiratory, and h circulatory causes with 99th percentile HNe (28.9 °C**⋅**h)**. HNe was modelled as a nonlinear continuous variable in the crossbasis function. All models were adjusted for trend, seasonality, weekdays, holidays, and daily levels of environmental covariates including mean temperatures averaging over lag 0–3 days, RH, total rainfall, wind speed, and PM_2.5_. Abbreviations: NCNE, non-cancer non-external; RH, relative humidity; PM_2.5_, fine particulate matter; HNe, hot night excess; SES, socioeconomic status.

During the hot season (May–October) of 2000–19, 5,002,144 NCNE hospitalizations were recorded, with over half in elderly (≥65 years) and about a quarter in the low SES group, and 18.5% and 12.8% due to respiratory and circulatory diseases, respectively (Table 1). The summer mean temperatures ranged from 18.7 °C to 32.4 °C (mean = 27.7 °C). During the 3680 study days, about half experienced excess nighttime heat, with the largest HNe at 50.7 °C⋅h; the number of hot nights identified by HNday_28 °C_ and HNday_90th_ were 499 (13.6%) and 187 (5.1%), respectively (Table 2). Due to the differing time frames between HNday_28 °C_ (00:00–23:59 of D_t_) and HNe (20:00 of D_t-1_–07:59 of D_t_), the two metrics do not always coincide (Fig. 1, Fig. 2). HNday_28 °C_ were characterized by varying intensities of HNe (mean = 14.3 °C⋅h), whereas HNday_90th_ were uniformly intense (mean = 22.1 °C⋅h) and captured 65 extra days with high HNe (17.9–50.7 °C⋅h) (eFigure 2). The occurrence of HNday_28 °C_ and HNday_90th_ peaked through late June to early August, gradually decreasing thereafter and became infrequent in October (eFigure 3). Importantly, over the past two decades we found a notable increase not only in the number of days with HNe >0 or hot nights as defined by HNday_28 °C_ and HNday_90th_, but also the occurrence of multiple consecutive hot nights, particularly since 2014 (eFigure 4).

**Table 1:** Summary statistics of daily counts of hospitalizations and levels of environmental exposure in Hong Kong, 2000–2019 (May–October).

Variable	N^a^	Mean	SD	Percentiles of distribution				
				Min	25th	50th	75th	Max
**Hospitalization**								
NCNE								
**All**	**5,002,114**	**1359**	**271**	**589**	**1149**	**1323**	**1557**	**2164**
0–14 years	501,612	136	34	27	114	135	157	290
15–64 years	1,926,658	524	102	237	445	507	600	819
≥65 years	2,573,885	699	153	310	574	686	809	1122
High SES	3,738,762	1016	188	416	875	1023	1143	1673
Low SES	1,263,352	343	137	48	282	342	380	781
Respiratory	925,195	251	57	92	213	245	280	608
Circulatory	641,351	174	32	66	150	172	197	288
**Environmental exposure**								
Temperature (°C)								
Mean	3680	27.7	2.0	18.7	26.4	28.0	29.3	32.4
Minimum	3680	25.8	1.9	15.7	24.6	26.0	27.3	30.0
Maximum	3680	30.3	2.4	20.4	28.7	30.5	32.1	36.6
RH (%)	3680	79.7	8.3	33.0	75.0	80.0	85.0	99.0
Total rainfall (mm)	3680	11.1	27.0	0.0	0.0	0.0	8.3	307.1
Wind speed (km/h)	3680	21.3	10.4	3.0	13.5	19.6	27.3	102.1
PM_2.5_ (μg/m^3^)	3680	25.3	18.4	4.3	12.4	18.5	32.3	137.7
NO_2_ (μg/m^3^)	3680	46.6	17.7	4.1	33.6	42.9	55.6	128.1
O_3_ (μg/m^3^)	3680	37.8	25.0	5.2	18.4	28.3	52.3	164.9
SO_2_ (μg/m^3^)	3680	15.0	12.3	2.9	7.3	11.3	18.2	135.5
HNe (°C⋅h)								
All	3680	4.1	6.3	0.0	0.0	0.1	6.5	50.7
Among HNe>0 days	1874	8.0	6.8	0.1	2.3	6.3	12.3	50.7
Among HNday_28 °C_	499	14.3	5.6	2.2	10.4	13.6	17.6	38.2

Abbreviations: NCNE, non-cancer non-external; RH, relative humidity; PM_2.5_, fine particulate matter; NO_2_, nitrogen dioxide; O_3_, ozone; SO_2_, sulfur dioxide; HNe, hot night excess; HNday_28 °C_, hot night day with a daily minimum temperature of 28 °C; SES, socioeconomic status; SD, standard deviation.

a Sum of daily counts for hospitalization data and number of days for environmental exposure data.

**Table 2:** Summary statistics of non-HNday_90th_ and HNday_90th_ with different subtypes.

Variable	N^a^	HNe (°C⋅h)^b^	Mean temperature (°C)^b^	Minimum temperature (°C)^b^	Maximum temperature (°C)^b^
Non-HNday_90th_	3493	3.1 (4.7)	27.6 (1.9)	25.7 (1.9)	30.1 (2.3)
HNday_90th_	187	22.1 (5.2)	30.2 (0.9)	28.0 (1.4)	32.8 (1.2)
Length of consecutive HNday_90th_					
1 d	62	22.3 (6.6)	29.7 (0.9)	27.4 (1.6)	32.5 (1.1)
2–3 d	93	22.0 (4.5)	30.4 (0.8)	28.3 (1.3)	33.1 (1.3)
≥ 4 d	32	22.3 (3.9)	30.5 (0.5)	28.5 (1.1)	32.8 (1.1)
Occurrence of HNday_90th_ in the hot season^c^					
Early	44	20.6 (3.1)	30.1 (0.7)	28.2 (1.4)	32.8 (1.3)
Middle	127	22.1 (4.8)	30.2 (0.8)	28.1 (1.3)	32.9 (1.3)
Late	16	27.0 (9.0)	29.6 (1.3)	27.1 (1.8)	32.6 (1.1)

Abbreviations: SD, standard deviation; HNe, hot night excess; HNday_90th_, hot night day with a minimal HNe of 17.7 °C⋅h (90th percentile).

a Number of days.

b Mean (SD) were calculated for each variable.

c Early, middle, and late hot seasons were defined as May–June, July–August, and September–October, respectively.

### Hospitalization hazards associated with HNday_28 °C_ and HNe

After adjusting the main effect of ambient mean temperature, HNday_28 °C_ showed no overall association with hospitalizations over cumulative lag 0–4 days (Table 3). In contrast, each 10 °C⋅h increase in HNe was associated with 1.9% [95% CIs: 0.2%, 3.6%] higher risk of hospitalization, and days with 99th percentile of HNe (28.9 °C⋅h) had 3.1% (1.5%, 4.8%) higher risk compared to days with zero HNe. The ERRs associated with HNe were particularly pronounced in elderly and low SES individuals (both Wald-test *p* value < 0.05). These associations were broadly consistent upon two-way stratification by age and SES groups (eTable 2). When restricted to specific causes of hospitalization, HNe was associated with significant ERR of circulatory (4.3% [1.8%, 6.9%] per 10 °C⋅h) but not respiratory disease (Table 3).

**Table 3:** Cumulative excess relative risks (95% confidence interval) over lag 0–4 days of hospitalizations associated with HNday_28 °C_ and HNe.

Hospitalization	HNday_28 °C_		HNe			
	HNday_28 °C_ days vs non-HNday_28 °C_ days^a^	Wald-test *p* value^d^	99th percentile vs 0 °C⋅h^b^	Wald-test *p* value^d^	Per 10 °C⋅h increase^c^	Wald-test *p* value^d^
NCNE						
All	−0.2 (−1.2, 0.7)	NA	**3.1 (1.5, 4.8)**	NA	**1.9 (0.2, 3.5)**	NA
0–14 years	**−2.9 (−5.4, −0.3)**	0.121	−0.1 (−4.2, 4.2)	0.028	−0.7 (−6.1, 5.0)	0.205
15–64 years	0.3 (−0.9, 1.5)	0.266	1.4 (−0.9, 3.7)	0.015	0.7 (−1.6, 3.0)	0.103
≥65 years	−0.7 (−1.9, 0.6)	Reference	**5.3 (3.2, 7.4)**	Reference	**3.1 (1.3, 5.0)**	Reference
High SES	−0.2 (−1.0, 0.6)	0.406	**2.1 (0.4, 4.0)**	0.045	1.1 (−0.6, 2.8)	0.168
Low SES	−0.9 (−2.5, 0.7)	Reference	**5.3 (2.8, 8.0)**	Reference	**3.8 (0.3, 7.5)**	Reference
Respiratory	−0.0 (−1.6, 1.6)	NA	1.2 (−2.3, 4.8)	NA	1.8 (−0.6, 4.3)	NA
Circulatory	1.2 (−0.4, 2.9)	NA	**3.4 (0.2, 6.8)**	NA	**4.3 (1.8, 6.9)**	NA

Abbreviations: NCNE, non-cancer non-external; RH, relative humidity; PM_2.5_, fine particulate matter; HNe, hot night excess; HNday_28 °C_; hot night day with a daily minimum temperature of 28 °C; SES, socioeconomic status; NA, not applicable. **Bold** estimates: *p* value < 0.05.

a HNday_28 °C_ was modelled as an indicator variable in the crossbasis function.

b HNe was modelled as a nonlinear continuous variable in the crossbasis function. HNe at the 99th %tile was 28.9 °C⋅h.

c HNe was modelled as a linear continuous variable in the crossbasis function.

d Wald tests were performed to test the differences in ERRs between NCNE age (reference: ≥65 years) and SES (reference: low SES) groups. All models were adjusted for trend, seasonality, weekdays, holidays, and daily levels of environmental covariates including mean temperatures averaging over lag 0–3 days, RH, total rainfall, wind speed, and PM_2.5_.

Regarding the shape of the cumulative (lag 0–4 days) exposure–response relationship of HNe with hospitalization, the overall association shows a marginal (<1%) dip of ERR at around 10 °C⋅h and an almost linear increased risk above 21.8 °C⋅h (Fig. 1). The threshold of significant excess risk in elderly emerged at a lower HNe (17.8 °C⋅h), whereas the associations in younger individuals were largely non-significant. Both SES groups showed significant ERRs upon high NHe exposure, but the low SES group had a lower threshold and much steeper rise of hospitalization risk than the high SES group. The apparent threshold for circulatory disease appeared even lower at ∼5.5 °C⋅h.

We examined the lag-response associations at 99th percentile of HNe (28.9 °C⋅h) and found significant ERR on lag 0, followed by decreased risk on lag 1, a typical phenomenon known as displacement or harvesting (Fig. 2).^8^ Some evidence of excess risk was also found on 2–3 days lag, particularly in elderly and for circulatory disease. In contrast, the significant ERR in the low SES group appeared to be driven primarily by a same-day association.

### Hospitalization hazards associated with HNday_90th_ and its subtypes

HNday_90th_ was associated with 1.9% [0.7%, 3.0%] higher risk of hospitalization in the overall population (Table 4). This association persisted and appeared somewhat stronger in some subgroups, particularly the elderly, low SES group, and circulatory hospitalizations. Interestingly, HNday_90th_ that lasted for 1 day and ≥4 days were associated with significant ERRs of hospitalization, but not those lasted for 2–3 days, probably due to inclusion of harvesting periods. The associations across subgroups of age, SES and causes of hospitalization were similar to the overall analysis. There is also suggestive evidence showing stronger association for HNday_90th_ that occurred during the late-hot season (i.e., September and October), with an overall excess risk of 7.2% [3.5%, 11.1%].

**Table 4:** Cumulative excess relative risks (95% confidence interval) over lag 0–4 days of hospitalizations associated with HNday_90th_ and its subtypes.

Hospitalization	All HNday_90th_ (n = 187)	Length of consecutive HNday_90th_			Occurrence of HNday_90th_ in the hot season^a^		
		1 d (n = 62)	2–3 d (n = 93)	≥4 d (n = 32)	Early (n = 44)	Middle (n = 127)	Late (n = 16)
NCNE							
All	**1.9 (0.7, 3.0)**	**3.9 (1.4, 6.5)**	0.4 (−1.1, 1.8)	**3.1 (1.2, 5.0)**	1.9 (−0.2, 4.1)	1.0 (−0.3, 2.4)	**7.2 (3.5, 11.0)**
0–14 years	0.6 (−2.3, 3.5)	−1.0 (−6.9, 5.2)	−2.7 (−6.3, 1.0)	4.7 (−0.1, 9.7)	−1.5 (−6.7, 3.9)	0.2 (−3.2, 3.6)	8.7 (−0.7, 18.8)
15–64 years	0.2 (−1.3, 1.7)	1.3 (−1.9, 4.6)	−0.6 (−2.6, 1.5)	0.9 (−1.7, 3.6)	−0.5 (−3.1, 2.3)	−0.4 (−2.1, 1.3)	**8.6 (3.7, 13.8)**
≥ 65 years	**2.8 (1.4, 4.1)**	**6.3 (3.1, 9.5)**	1.3 (−0.4, 3.1)	**3.4 (1.1, 5.7)**	2.5 (−0.1, 5.1)	**2.3 (0.7, 3.9)**	**4.6 (0.2, 9.2)**
High SES	**1.6 (0.3, 2.9)**	**3.3 (0.6, 6.1)**	−0.0 (−1.6, 1.6)	**2.9 (0.8, 5.0)**	1.8 (−0.5, 4.2)	0.7 (−0.8, 2.2)	**6.5 (2.4, 10.6)**
Low SES	**2.1 (0.4, 3.8)**	**4.3 (0.4, 8.3)**	0.8 (−1.3, 3.0)	**3.0 (0.3, 5.7)**	3.0 (−0.2, 6.2)	1.1 (−0.9, 3.1)	**10.4 (4.7, 16.5)**
Respiratory	1.7 (−0.5, 3.8)	1.6 (−3.1, 6.5)	0.1 (−2.6, 2.8)	3.6 (−0.0, 7.3)	2.1 (−1.8, 6.2)	1.0 (−1.4, 3.4)	1.0 (−5.5, 7.9)
Circulatory	**2.3 (0.1, 4.7)**	5.1 (−0.0, 10.4)	1.1 (−1.8, 4.1)	**4.3 (0.4, 8.3)**	**4.7 (0.4, 9.1)**	1.5 (−1.1, 4.2)	3.7 (−3.3, 11.3)

Abbreviations: HNday_90th_, hot night day with a minimal HNe of 17.7 °C⋅h (90th percentile); NCNE, non-cancer non-external; SES, socioeconomic status. **Bold** estimates: *p* value < 0.05.

a Early, middle, and late hot seasons were defined as May–June, July–August, and September–October, respectively. HNday_90th_, its length, and occurrence in the hot season were modelled as indicator variables in the crossbasis function individually. All models were adjusted for trend, seasonality, weekdays, holidays, and daily levels of environmental covariates including mean temperatures averaging over lag 0–3 days, RH, total rainfall, wind speed, and PM_2.5_. All estimates were in reference to non-HNday_90th_ days (n = 3493).

### Sensitivity analyses

The associations between NCNE hospitalizations and HNe were largely consistent across sensitivity analyses, including those with: i) increased maximum lag, ii) increased number of knots in the crossbasis function of HNe, iii) daily mean temperature fitted as a crossbasis function, iv) fewer *df* for temporal variation, and v) adjustment for other pollutants (eTable 3). The effect estimates from HNe calculated from sunset and sunrise time were largely consistent with the present findings (eFigure 6).

## Discussion

The present study found HNe and intensive hot nights (HNday_90th_), but not the government-defined HNday_28 °C_, to be associated with significant excess risk of NCNE hospitalization in a densely populated subtropical metropolis, with considerable disparities by age and SES. In particular, the associations of HNe and HNday_90th_ with hospitalization were particularly pronounced in elderly aged 65+ years and individuals of low SES.

To the best of our knowledge, this is the first study examining the association of excess nighttime heat with hospitalization risk in a subtropical city, building upon prior research that focused on mortality risks mostly in populations in temperate regions.^9^^,^^10^^,^^14^, ^15^, ^16^ In particular, prior research has found that, compared to no HNe, non-accidental mortality increased by 12–37% at extreme HNe (∼32–72 °C⋅h) in southern European countries (Spain, Portugal, France, Italy) and by 30–50% at extreme HNe (∼35–38 °C⋅h) in Asian countries (China, Japan, South Korea).^9^^,^^10^ Our findings cannot be directly compared to those from mortality studies due to the use of different outcomes, and few studies have investigated morbidity outcomes. Notably, a recent hospital-based study in 15 Chinese cities found ∼10 °C⋅h HNe to be associated with 14% [95% CI: 7%, 22%] higher risk of outpatient events due to circulatory disease,^12^ but another study in urban Shanghai, China found little evidence of association.^29^ In the case of hot-night days, an earlier study in Hong Kong found 2.4–2.7% higher risk of natural deaths during hot nights, similarly defined as HNday_28 °C_, with females and the elderly being more affected.^15^ It also suggested that higher mortality hazards arose from ≥5 consecutive hot nights.^15^

In our study conducted in subtropical Hong Kong, the average ERR of hospitalization associated with each 10 °C⋅h HNe observed were relatively small, amounting to 1.9% in the general population and 3–5% for elderly, socioeconomically disadvantaged individuals, and circulatory hospitalizations. While the ERR per 10 °C⋅h higher HNe represents an easily comprehensible and policy-relevant metric for the averaged strength of association across the entire exposure range, the HNe-hospitalization association was found to be non-linear. Specifically, significant excess risks emerged at 15–20 °C⋅h, suggesting a potential threshold effect, with lower thresholds in vulnerable subgroups and for circulatory hospitalization, which is likely most sensitive to the effects of nighttime heat, compared to other diseases. For hot-night days, we observed significantly higher NCNE hospitalization risks only during HNday_90th_, particularly for elderly, low SES individuals, and HNday_90th_ that lasted for ≥4 consecutive nights, but not during HNday_28 °C_.

Our findings of generally mild hospitalization hazards related to excess nighttime heat and hot nights are likely multi-factorial. First, previous studies that examined mortality were likely to capture a relatively strong harvesting effects among vulnerable populations (e.g., multi-morbid individuals, elderly), whereas morbidity studies tend to show weaker associations due to dilution from less sensitive/relevant illnesses and missing or delayed cases in the hospitalization records, especially in the working-age population who likely prioritize work over immediate medical attention.^30^ Although we also observed signs of harvesting, it appeared to be more transient, with significant excess risks observed at lag 0 and lag 2–3 in general but an apparent lower risk at lag 1, when the background risk of the population may be reduced after the most vulnerable individuals were hospitalized at lag 0. Second, people living in hotter areas, such as Hong Kong, tend to have greater capacity to mitigate the effect of heat through resilient infrastructure (e.g., high air-conditioning [AC] prevalence) and physiological acclimatization, leading to higher thresholds for developing heat-related illnesses, compared to those living in temperate climates.^8^ In fact, previous studies on daily mean temperature have also found weak to no association between heat and hospitalization in Spanish populations,^13^^,^^31^ whereas the heat-mortality association has been consistently reported across populations. Compared to previous studies in other populations, Hong Kong is known to have a relatively healthy population with some of the world's highest life expectancy, a highly cost-efficient healthcare system, and a highly urbanized environment with high air-conditioning ownership. All these factors may have contributed to dampen the adverse effects of nighttime heat in Hong Kong. This is coherent with the apparent non-linear, threshold effect suggesting the population could adapt to mild HNe, and excessive HNe would overwhelm individuals' ability to adapt (both biologically and behaviorally).

Our study has demonstrated noticeable adverse associations of HNe and HNday_90th_ with hospitalizations, indicating the importance of considering nighttime heat intensity in defining hot nights relevant to public health. In the study population, HNday_28 °C_ encapsulated a wide range of HNe, including what was well below the apparent threshold of elevated hospitalization risk. Moreover, since air temperatures gradually decrease after sunset and often reach the lowest point around or after sunrise, solely relying on a daily minimum temperature of 28 °C may overlook days that are hotter in the early night but cool down later with temperatures falling below 28 °C. This is especially concerning because prior research suggested that excess heat in the early night is more likely to disturb sleep onset and may be more harmful.^9^ Our findings call for a careful review of the current governmental definition of hot-night days and the associated public warning system and adaptation empowerment strategies, which should consider the intensity of nighttime heat instead of a single cut-off.

Importantly, we have shown how HNe disproportionately affects low-SES individuals. Growing evidence suggests that socioeconomic disadvantages, such as residence in deprived communities, lower income levels, and social isolation, increase individuals’ vulnerability to heat.^17^^,^^22^ Hong Kong endures a strong UHI effect that causes hotter summer nights in densely-populated districts (e.g., Sham Shui Po), where greater proportion of socioeconomically-deprived and vulnerable individuals reside, compared to the sub-urban areas.^21^ Moreover, despite the high AC prevalence in Hong Kong, low-SES individuals may be less likely to afford the installation or electricity costs necessary for adequate cooling to mitigate excess nighttime heat.

Our study also found elderly to be particularly vulnerable to the adverse effect of nighttime heat, and elderly of low-SES were the most susceptible group. Physiologically, aging leads to a decline in thermoregulation capabilities, and elderly with chronic comorbidities or those taking specific medications may exhibit increased sensitivity to non-optimal temperature.^7^ Some elderly opt for fans instead of ACs for cooling at night, as they worry about catching a cold from using ACs.^32^ However, in housing units with small windows and poor ventilation, which are common in Hong Kong, fans are likely ineffective in combating heat.^33^ Additionally, in Hong Kong, 13% of elderly live alone, constituting extra vulnerabilities due to lack of social or familial support,^34^ such as delayed medical care that could lead to severe health outcomes such as out-of-the-hospital fatalities. On the other hand, although working-age individuals are less vulnerable to extreme heat, we found those of low SES also experience increased HNe-related risks. This could be related to their employment in labor-intensive industries (e.g., construction, catering), where hot nights can hinder sleep and rest, resulting in inadequate relief and recovery from daytime labor and heat exposure.^34^

There are several strengths in the current study. First, we utilized an extended time-series dataset spanning 20 years, which provides relatively robust statistical power. Second, we ascertained the effect of hot-night metrics independent of average temperatures by adjusting for multi-day averages. Third, while most previous studies relied on district-level socioeconomic indices for SES classification, we employed a well-established individual-level indicator. However, some limitations warrant discussion. First, as in most previous aggregated time-series analyses, we relied on meteorological data from fixed weather stations to represent the city-wide average heat exposure and it may not accurately capture personally-experienced temperature in the participants. Second, our study did not include hospitalizations due to external causes (e.g., accidents and suicide), which have been reported to be sensitive to heat in recent literature.^35^ Third, despite the long-term, city-wide data, some subgroups (e.g., young children aged 0–14 years) recorded relatively limited cases and hampered the statistical power of related analyses, the findings from which should be interpreted cautiously. Fourth, although we have found suggestive evidence of greater ERR in relation to HNday_90th_ in the late-hot season, which might reflect how unusual nighttime heat in prolonged hot season overwhelmed adaptation capacity, the small number of HNday_90th_ (n = 16) in this group and the ecological design prevented us from directly evaluating the mitigation effects of adaptation or acclimatization. Further studies with individual-level data are needed to clarify that.

Our study provides new evidence of increased hospitalization risks in relation to excessive nighttime heat and hot nights in a highly-densely populated, subtropical urban setting. Socioeconomically disadvantaged individuals, particularly elderly, appeared more vulnerable to nighttime heat. It is crucial for society to increase the awareness of the potential harm of hot nights and enhance adaptation in response to the heating climate. For policymakers, the intensity of nighttime heat should be considered when defining hot nights in heat-warning systems.

## Contributors

**Yi Tong Guo**: Conceptualization, Methodology, Formal analysis, Data access & verification, Writing–Original Draft, Visualization. **Ka Hung Chan**: Conceptualization, Methodology, Writing–Review & Editing, Supervision. **Qiu Hong**: Writing–Review & Editing. **Eliza Lai-yi Wong**: Supervision, Project administration. **Kin-fai Ho**: Conceptualization, Resources, Data access & verification, Writing–Review & Editing, Supervision, Project administration, funding acquisition.

## Data sharing statement

The data of hospital admissions are available from Hong Kong Hospital Authority but restrictions apply to the availability of these data, which were used under license for the current study, and so are not publicly available. The data of meteorological variables are available in the Hong Kong Observatory open database https://www.hko.gov.hk/en/abouthko/opendata_intro.htm. The data of air pollutants are available in the Hong Kong Environmental Protection Department open database https://cd.epic.epd.gov.hk/EPICDI/air/station/?lang=en.

## Ethical approval and consent to participate

As our study used aggregated data and did not involve individuals directly, informed consent was not obtained from the patients and ethical approval was exempt.

## Declaration of interests

The authors declare that they have no known competing financial interests or personal relationships that could have appeared to influence the work reported in this paper.
