# Supplementary material for: The risk of hospitalization associated with hot nights and excess nighttime heat in a subtropical metropolis: a time-series study in Hong Kong, 2000–2019
Source: Lancet Reg Health West Pac. 2024 Aug 12;51:101168. doi: 10.1016/j.lanwpc.2024.101168 (PMC11367509; doi:10.1016/j.lanwpc.2024.101168)
Supplement: Supplementary materials [file mmc1.docx]

***Supplementary materials***

**The risk of hospitalization associated with hot nights and excess nighttime heat in a subtropical metropolis: a time-series study in Hong Kong, 2000-2019**

Yi Tong GUO^a^, Ka Hung CHAN^b,*^, Hong QIU^a^, Eliza Lai-yi WONG^a^, Kin Fai HO^a,c,**^

^a^ JC School of Public Health and Primary Care, The Chinese University of Hong Kong, HKSAR, China.

^b^ Clinical Trial Service Unit and Epidemiological Studies Unit, Nuffield Department of Population Health, University of Oxford, Oxford, UK.

^c^ Institute of Environment, Energy and Sustainability, The Chinese University of Hong Kong, HKSAR, China.

^*^ Corresponding author. Clinical Trial Service Unit and Epidemiological Studies Unit, Nuffield Department of Population Health, University of Oxford, Oxford, UK. E-mail address: [kahung.chan@ndph.ox.ac.uk](mailto:kahung.chan@ndph.ox.ac.uk) (KH Chan).

^**^ Corresponding author. JC School of Public Health and Primary Care, The Chinese University of Hong Kong, Shatin, New Territories, HKSAR, China. E-mail address: [kfho@cuhk.edu.hk](mailto:kfho@cuhk.edu.hk) (KF Ho).

**Tables of eEquations, eFigures, and eTables**

[eEquation 1 3](#_Toc165045318)

[eEquation 2 3](#_Toc165045319)

[eFigure 1 Temporal variation of hourly temperatures during nighttime hours among HNe>0 days (N=1874). 4](#_Toc165044479)

[eFigure 2 Temporal variation of hourly temperatures of days with extremely high HNe but were not identified as HNday28°C. 5](#_Toc165044480)

[eFigure 3 Trend and seasonal distribution of HNe>0 days, HNday28°C, and HNday90th over 2000-2019 (May to Oct). 6](#_Toc165044481)

[eFigure 4 Occurrence of HNday90th over 2000-2019 (May to Oct). 7](#_Toc165044482)

[eFigure 5 Time-series plots of daily counts of hospitalizations in Hong Kong, 2000-2019 (May - Oct). 9](#_Toc165044483)

[eFigure 6. Cumulative exposure-response associations (lag 0-4 days) of hospitalizations with HNe based on sunset and sunrise time in Hong Kong, 2000-2019 (May to Oct). 10](#_Toc169401109)

[eTable 1 Summary statistics of daily counts of age-SES-specific NCNE hospitalizations. 11](#_Toc165044462)

[eTable 2 Cumulative excess relative risks (95% confidence interval) over lag 0-4 days of age-SES-specific NCNE hospitalizations associated with HNday28°C and HNe. 12](#_Toc165044463)

[eTable 3 Sensitivity results of cumulative excess relative risks (95% confidence intervals) of all NCNE hospitalizations associated with HNe. 13](#_Toc165044464)

**Supplementary methods 1**

The eEquation (1) for decomposition is shown as below:

| $\begin{matrix} Y_{t}\sim quasiPoisson\left( \mu_{t} \right) \\ log\left( \mu_{t} \right)=\beta_{0}+s\left( DOS_{t},k=1-6/year \right)+\beta_{3}DOW_{t}+\beta_{4}Holiday_{t}+ \\ \beta_{5}Shift_{t}+\beta_{6}\mu_{t-1}+\beta_{7}\mu_{t-2}+\epsilon_{t} \end{matrix}$ | eEquation 1 |
| --- | --- |

The eEquation (2) for full regression is shown as below:

| $\begin{matrix} log\left( \mu_{t} \right)=\beta_{0}+s\left( DOS_{t},k=1-6/year \right)+\beta_{3}DOW_{t}+\beta_{4}Holiday_{t}+ \\ \beta_{5}Shift_{t}+\beta_{6}\mu_{t-1}+\beta_{7}\mu_{t-2}+s\left( tmean{03}_{t} \right)+s\left( RH_{t} \right) \\ +s\left( wind_{t} \right)+s\left( rain_{t} \right)+s\left( PM_{2.5t} \right)+\beta_{8}cb_{t,l}+\epsilon_{t} \end{matrix}$ | eEquation 2 |
| --- | --- |

Here, generalized additive models (GAM) were applied assuming a quasi-Poisson distribution to allow for overdispersion in the count series.[^1^](#ref-2006Wood) $\mu_{t}$ referred to the expected number of hospitalizations on day $t$. A crossbasis function ($cb_{t,l}$) with a maximum lag ($l$) of 7 days was created for each hot-night metric using DLNMs, which forms a bi-dimensional matrix integrating the exposure-response and lag-response associations and allows for nonlinearity.[^2^](#ref-2010GasparriniaArmstrong) Specifically, for the exposure dimension, linear functions and natural cubic splines with one knot placed at the 50^th^ percentile of the distribution were fit for HNe, while indicator functions were fit for HNday_28°C_, and HNday_90th_ and its subtypes, respectively; for the lag dimension, quadratic B-splines with two knots equally placed on the log scale of the lag range were applied for all hot-night metrics. Additionally, $s\left( DOS,k=1-6/year \right)$ referred to thin-plate splines ($s\left( . \right)$) of day of study ($DOS_{t}$) with the number of basis ($k$) ranging from 1-6/year to control for different variation in the outcomes. $DOW_{t}$ and $Holiday_{t}$ were indicators for day of week and public holidays, respectively, to control for potential weekday and holiday patterns. As there has been an expansion of medical fee waiver scheme since Jul 15, 2017, the daily counts of hospitalizations in SES-specific groups significantly changed afterwards. For a handy adjustment, we added an indicator for the level shift ($Shift_{t}$) for all outcome series, with 0 for days before Jul 15, 2017 and 1 for the rest days. Furthermore, auto-regression terms ($AR$) by lagging the outcome series for 1 day and/or 2 days ($\mu_{t-1}$ and $\mu_{t-2}$) were added to control for residual autocorrelation if necessary. The choices of $k$ (i.e., 1-6/year), whether including $AR$ or the number of $AR$ (i.e., 1 or 2) were determined by examining the partial autocorrelation (PAC) plots and minimizing quasi-Akaike’s Information Criterion (qAIC) scores.

The models were adjusted for environmental covariates, including same-day RH ($RH_{t}$), total rainfall ($rain_{t}$), wind speed ($wind_{t}$), PM_2.5_ ($PM{2.5}_{t}$), and lag 0-3 moving average mean temperature ($tmean{03}_{t}$). Prior time-series studies have found RH and wind speed might affect perceived temperatures and were associated with mortality outcomes.[^3^](#ref-2019ArmstrongSera)^,^[^4^](#ref-2012WichmannKetzel) Therefore, they are often considered as confounders in temperature epidemiology studies. We controlled for same-day rainfall based on the assumption that heavy rainfall might prevent some patients with less sever medical conditions from visiting hospitals and alter their time spent outdoors.[^5^](#ref-2017GogginsChan) PM_2.5_ is widely reported as a risk factor for adverse health outcomes[^6^](#ref-2014AtkinsonKang) and is known to be correlated with temperature, so it was adjusted in the present study.


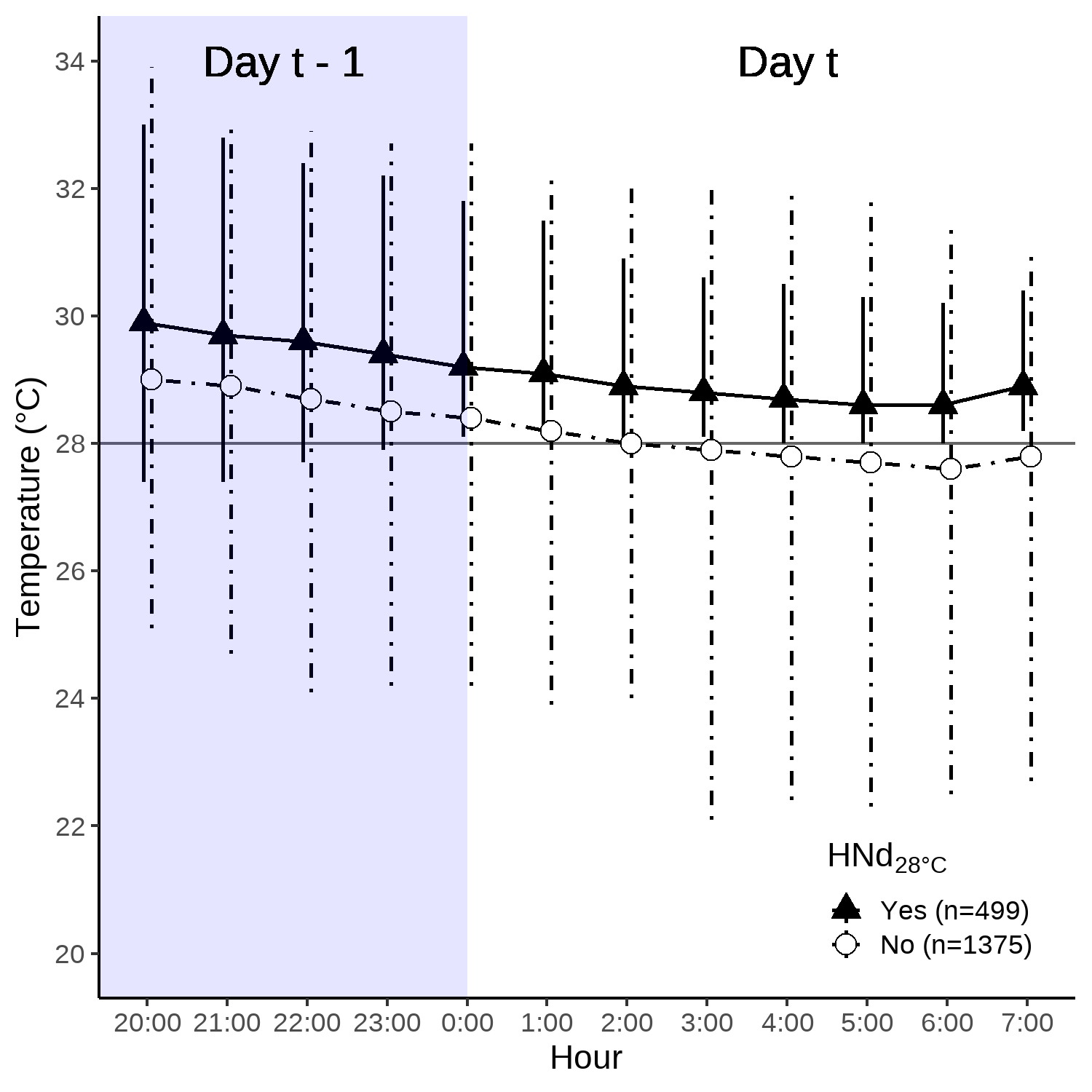


eFigure 1 Temporal variation of hourly temperatures during nighttime hours among HNe>0 days (N=1874).

Nighttime hours refer to the 12-h period from 20:00 of the previous calendar day (Day t-1) to 7:59 of the current calendar day (Day t). The points and line ranges refer to the median and range of hourly temperatures, respectively. Abbreviation: HNe, hot night excess; HNday_28°C_, hot night day with a daily minimum temperature of 28 °C.


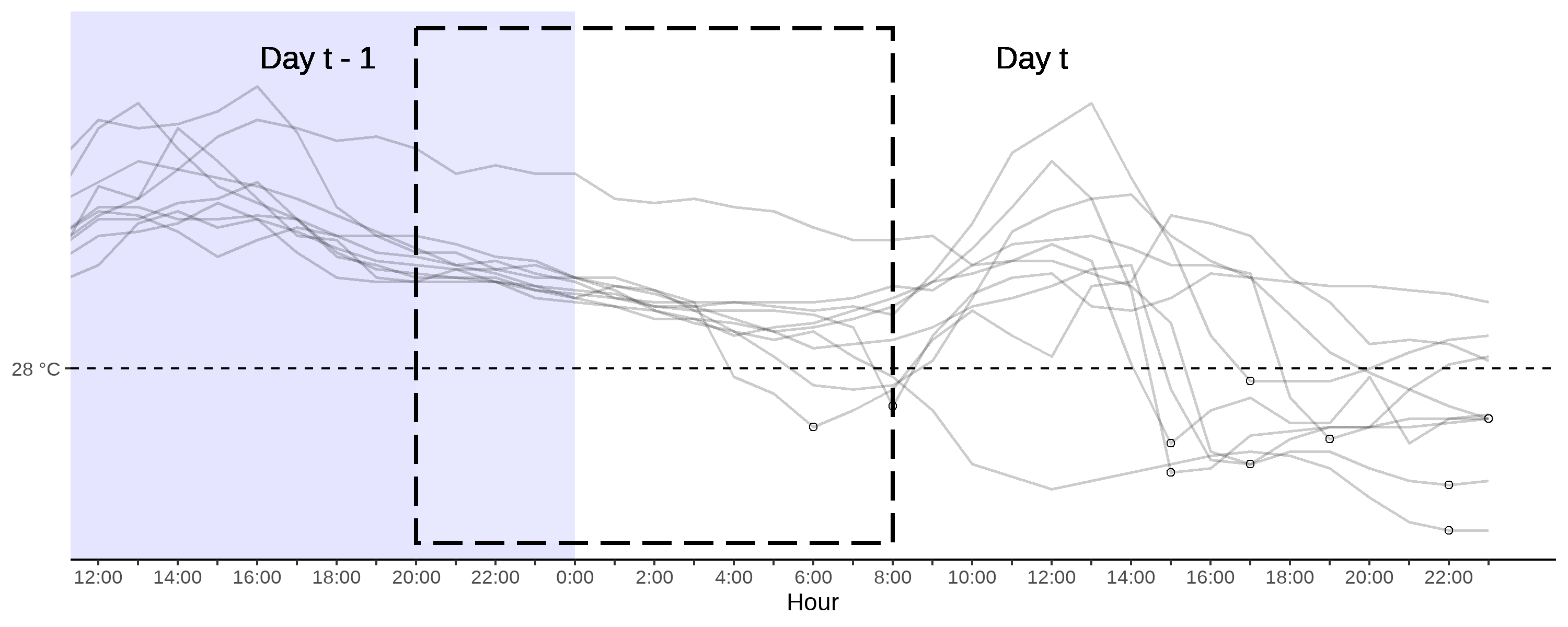


eFigure 2 Temporal variation of hourly temperatures of days with extremely high HNe but were not identified as HNday_28°C_.

Ten out of 65 days were shown as examples. The dashed rectangle frame denoted nighttime hours from 20:00 of Day_t-1_ to 7:59 of Day_t_. The void circle denoted the occurrence of minimum temperature on Day_t_. Abbreviations: HNe, hot night excess; HNday_28°C_, hot night day with a daily minimum temperature of 28 °C.


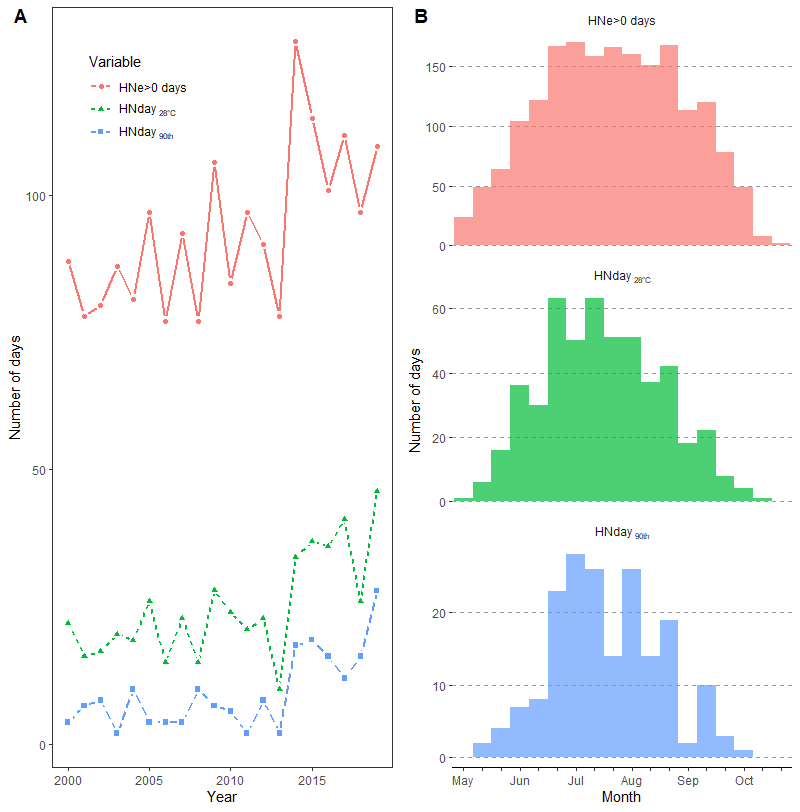


eFigure 3 Trend and seasonal distribution of HNe>0 days, HNday_28°C_, and HNday_90th_ over 2000-2019 (May to Oct).

Abbreviations: HNe, hot night excess; HNday_28°C_, hot night day with a daily minimum temperature of 28 °C; HNday_90th_, hot night day with a minimal HNe of 17.7 °C·h (90^th^ percentile).


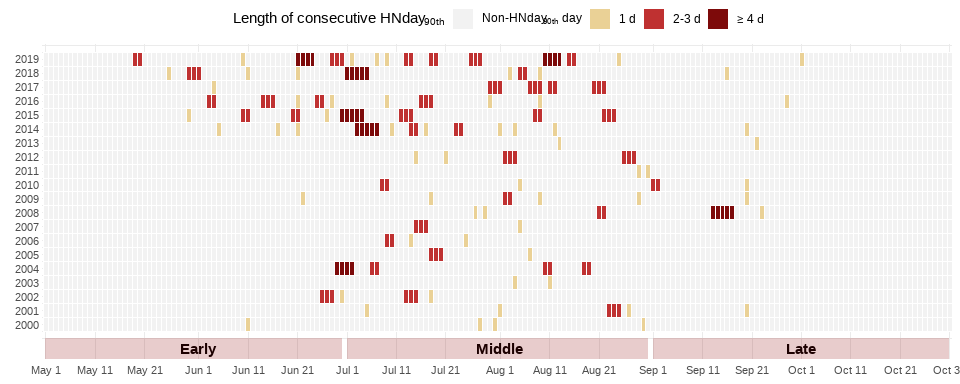


eFigure 4 Occurrence of HNday90th over 2000-2019 (May to Oct).

Abbreviations: HNday_90th_, hot night day with a minimal HNe of 17.7 °C·h (90^th^ percentile).


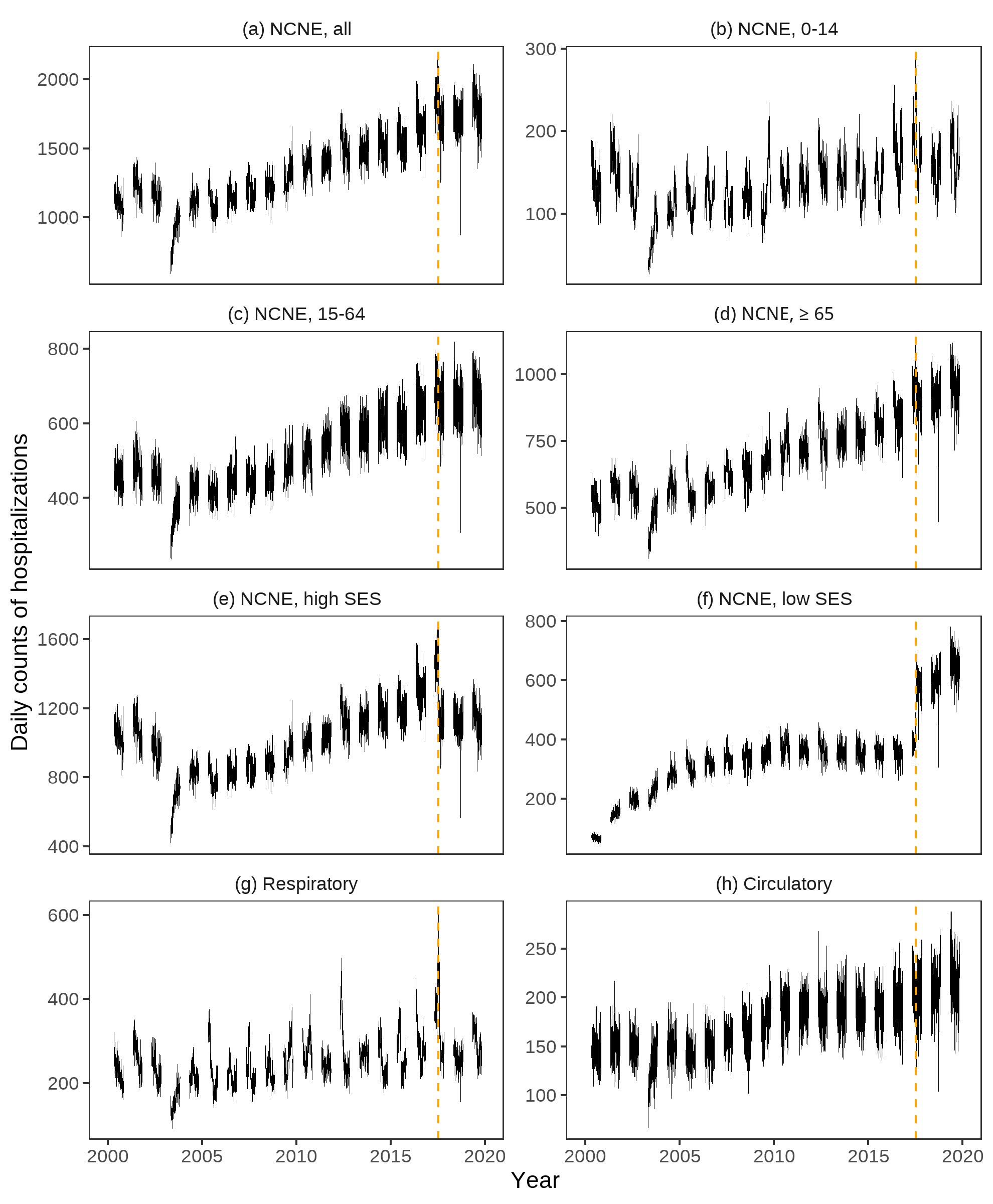


eFigure 5 Time-series plots of daily counts of hospitalizations in Hong Kong, 2000-2019 (May - Oct).

The orange dashed vertical line indicated 2017-07-15 when the policy of expanding public assistance eligibility launched, leading to level shifts in SES groups. Abbreviations: NCNE, non-cancer non-external; SES, socioeconomic status.


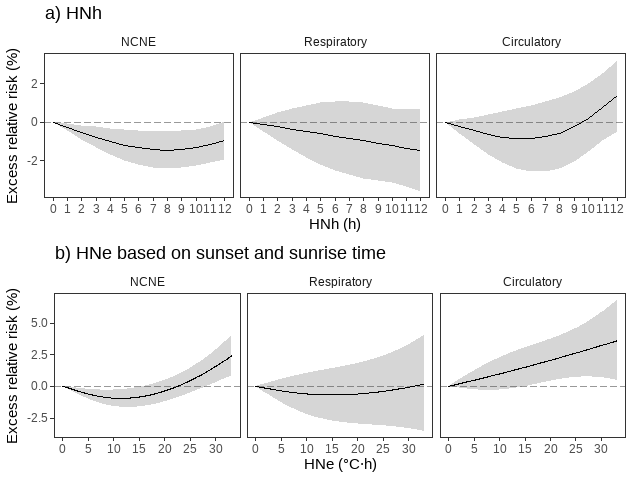


eFigure 6. Cumulative exposure-response associations (lag 0-4 days) of hospitalizations with HNe based on sunset and sunrise time in Hong Kong, 2000-2019 (May to Oct).

HNe was modelled as nonlinear continuous variable in the crossbasis function. All models were adjusted for trend, seasonality, weekdays, holidays, and daily levels of environmental covariates including mean temperatures averaging over lag 0-3 days, RH, total rainfall, wind speed, and PM_2.5_. Abbreviations: NCNE, non-cancer non-external; RH, relative humidity; PM_2.5_, fine particulate matter; HNe, hot night excess; SES, socioeconomic status.

eTable 1. Summary statistics of daily counts of age-SES-specific NCNE hospitalizations.

| **Variable** | **N ^a^** | **Mean** | **SD** | **Percentiles of distribution** | | | | |
| --- | --- | --- | --- | --- | --- | --- | --- | --- |
|  |  |  |  | Min | 25^th^ | 50^th^ | 75^th^ | Max |
| NCNE | | | | | | | | |
| 0-14 years, high SES | 449 302 | 122 | 33 | 23 | 99 | 121 | 144 | 261 |
| 0-14 years, low SES | 52 310 | 14 | 6 | 0 | 10 | 14 | 18 | 46 |
| 15-64 years, high SES | 1 653 836 | 449 | 89 | 194 | 379 | 443 | 514 | 710 |
| 15-64 years, low SES | 272 822 | 74 | 25 | 4 | 63 | 80 | 91 | 125 |
| ≥ 65 years, high SES | 1 635 614 | 444 | 90 | 176 | 385 | 436 | 495 | 814 |
| ≥ 65 years, low SES | 938 271 | 255 | 117 | 33 | 199 | 243 | 272 | 649 |
| *Note:*  ^a^ Sum of daily counts for hospitalization data. Abbreviations: NCNE, non-cancer non-external; SES, socioeconomic status; SD, standard deviation. | | | | | | | | |

eTable 2. Cumulative excess relative risks (95% confidence interval) over lag 0-4 days of age-SES-specific NCNE hospitalizations associated with HNday_28°C_ and HNe.

| **Variable** | **HNday_28°C_** |  | **HNe** | |
| --- | --- | --- | --- | --- |
|  | HNday_28°C_ days v.s. non-HNday_28°C_ days ^a^ |  | 99^th^ percentile v.s. 0 °C·h ^b^ | Per 10 °C·h increase ^c^ |
| NCNE |  |  |  |  |
| 0-14 years, high SES | **-3.0 (-5.1, -0.9)** |  | -1.3 (-7.2, 5.0) | **-3.9 (-7.6, -0.0)** |
| 0-14 years, low SES | 2.9 (-2.5, 8.5) |  | 4.0 (-6.5, 15.8) | 1.9 (-7.5, 12.3) |
| 15-64 years, high SES | 0.2 (-1.0, 1.5) |  | 0.9 (-1.4, 3.3) | -0.0 (-2.3, 2.3) |
| 15-64 years, low SES | 1.7 (-0.4, 3.8) |  | 4.3 (-0.3, 9.1) | **4.8 (1.3, 8.5)** |
| ≥ 65 years, high SES | 0.1 (-1.0, 1.1) |  | **2.9 (0.5, 5.4)** | 1.7 (-0.1, 3.6) |
| ≥ 65 years, low SES | -1.3 (-3.1, 0.4) |  | **6.3 (3.4, 9.2)** | **3.4 (0.5, 6.4)** |
| *Note:*  ^a^ HNday_28°C_ was modelled as an indicator variable in the crossbasis function.  ^b^ HNe was modelled as a nonlinear continuous variable in the crossbasis function. HNe at the 99^th^ %tile was 28.9 °C·h.  ^c^ HNe was modelled as a linear continuous variable in the crossbasis function. All models were adjusted for trend, seasonality, weekdays, holidays, and daily levels of environmental covariates including mean temperatures averaging over lag 0-3 days, RH, total rainfall, wind speed, and PM_2.5_. Abbreviations: NCNE, non-cancer non-external; RH, relative humidity; PM_2.5_, fine particulate matter; HNe, hot night excess; HNd_28°C_; hot night day with a daily minimum temperature of 28 °C; SES, socioeconomic status. **Bold** estimates: *p* value < 0.05. | | | | |

eTable 3. Sensitivity results of cumulative excess relative risks (95% confidence intervals) of all NCNE hospitalizations associated with HNe.

| **Model** | **ERRs (95% CIs) ^b^** |
| --- | --- |
| Main ^a^ | **3.1 (1.5, 4.8)** |
| Sensitivity analyses |  |
| 1 Cumulative effect over lag 0-7 days | **3.0 (0.9, 5.2)** |
| 2 Use two knots for exposure dimension in the crossbasis function of HNe | **2.3 (0.4, 4.2)** |
| 3 Use a crossbasis function with a maximum lag of 7 days for daily mean temperatures | **3.6 (1.4, 5.8)** |
| 4 Use k=4/year for day of study | **2.8 (1.2, 4.4)** |
| 5 Adjust for NO_2_ | **3.2 (1.6, 4.9)** |
| 6 Adjust for O_3_ | **3.3 (1.6, 4.9)** |
| 7 Adjust for SO_2_ | **3.3 (1.6, 5.0)** |
| *Note:*  ^a^ HNe was modeled as a continuous variable in the crossbasis function with a maximum lag of 7 days, where natural cubic splines of one knot were used for the exposure dimension and B-splines with two knots equally placed in the log scale of lag range were used for the lag dimension. The main model was adjusted for multiple temporal and environmental covariates, including thin-plate plates with k=3/year for day of study, indicators for day of week, indicators for public holidays, indicators for level shift, a lag-1 autoregression term, thin-plate plates with k=5 for daily mean temperatures averaging over lag 0-3 days, same-day RH, square-root same-day total rainfall, square-root same-day wind speed, square-root same-day PM_2.5_, respectively.  ^b^ Cumulative effects over lag 0-4 days comparing HNe at the 99^th^ %tile (28.9°C·h) to 0 °C·h were reported, except for sensitivity analysis 1. Abbreviations: NCNE, non-cancer non-external; RH, relative humidity; PM_2.5_, fine particulate matter; NO_2_, nitrogen dioxide; O_3_, ozone; SO_2_, sulfur dioxide; HNe, hot night excess; ERR, excess relative risk; CI, confidence interval. **Bold** estimates: *p* value < 0.05. | |

**Reference**

1. Wood SN. [Generalized Additive Models: An Introduction with R](https://doi.org/10.1201/9781315370279). 2nd ed. Chapman and Hall, CRC. New York: Chapman and Hall/CRC; 2006.

2. Gasparrinia A, Armstrong B, Kenward MG. [Distributed lag non-linear models](https://doi.org/10.1002/sim.3940). Statistics in Medicine. 2010 Sep;29(21):2224–34.

3. Armstrong B, Sera F, Vicedo-Cabrera AM, Abrutzky R, Åström DO, Bell ML, et al. [The Role of Humidity in Associations of High Temperature with Mortality: A Multicountry, Multicity Study](https://doi.org/10.1289/EHP5430). Environmental Health Perspectives. 2019 Sep;127(9):097007.

4. Wichmann J, Ketzel M, Ellermann T, Loft S. [Apparent temperature and acute myocardial infarction hospital admissions in Copenhagen, Denmark: A case-crossover study.](https://doi.org/10.1186/1476-069X-11-19) Environmental health. 2012 Dec;11(1):19.

5. Goggins WB, Chan EY. [A study of the short-term associations between hospital admissions and mortality from heart failure and meteorological variables in Hong Kong: Weather and heart failure in Hong Kong](https://doi.org/10.1016/j.ijcard.2016.11.106). International Journal of Cardiology. 2017 Feb;228:537–42.

6. Atkinson RW, Kang S, Anderson HR, Mills IC, Walton HA. [Epidemiological time series studies of PM2.5 and daily mortality and hospital admissions: A systematic review and meta-analysis](https://doi.org/10.1136/thoraxjnl-2013-204492). Thorax. 2014 Jul;69(7):660–5.
